# Mapping health outcome measures from a stroke registry to EQ-5D weights

**DOI:** 10.1186/1477-7525-11-34

**Published:** 2013-03-07

**Authors:** Ola Ghatnekar, Marie Eriksson, Eva-Lotta Glader

**Affiliations:** 1 Department of Public Health and Clinical Medicine, Umeå University, Umeå, Sweden; 2 Department of Statistics, Umeå University, Umeå, Sweden

**Keywords:** Mapping, Quality of life, Stroke, Sweden, Transfer to utility, Utility

## Abstract

**Purpose:**

To map health outcome related variables from a national register, not part of any validated instrument, with EQ-5D weights among stroke patients.

**Methods:**

We used two cross-sectional data sets including patient characteristics, outcome variables and EQ-5D weights from the national Swedish stroke register. Three regression techniques were used on the estimation set (n = 272): ordinary least squares (OLS), Tobit, and censored least absolute deviation (CLAD). The regression coefficients for “dressing“, “toileting“, “mobility”, “mood”, “general health” and “proxy-responders” were applied to the validation set (n = 272), and the performance was analysed with mean absolute error (MAE) and mean square error (MSE).

**Results:**

The number of statistically significant coefficients varied by model, but all models generated consistent coefficients in terms of sign. Mean utility was underestimated in all models (least in OLS) and with lower variation (least in OLS) compared to the observed. The maximum attainable EQ-5D weight ranged from 0.90 (OLS) to 1.00 (Tobit and CLAD). Health states with utility weights <0.5 had greater errors than those with weights ≥0.5 (P < 0.01).

**Conclusion:**

This study indicates that it is possible to map non-validated health outcome measures from a stroke register into preference-based utilities to study the development of stroke care over time, and to compare with other conditions in terms of utility.

## Introduction

Stroke can be both a physically and mentally debilitating condition. This has implications on the intangible cost to the patient and care-givers in terms of substantially reduced health related quality of life (HRQoL) [1]. The development of stroke care is therefore important in order to alleviate some of this burden. As a means to such continuous quality improvement, national registers for acute stroke have been set up in several countries [2]. Data collection in these registers may include financial, clinical, process, and outcome indicators. The outcome indicators may include survival, satisfaction with health care services, and different patient related outcomes such as activities of daily living and HRQoL.

However, none of these outcome variables is suitable for making comparisons with improvements in other conditions unless it shares a common denominator for outcome evaluation. For example, mortality is an important indicator in stroke care but is of less importance for chronic conditions with low excess mortality. Generic preference-based measures of the HRQoL are today more or less common standards for such a common denominator. The advantage with these measures is that they reveal an individual’s preferences for one health state over another and therefore provide a cardinal scale (0.8 is better than 0.6). They are constructed from defined health states and tariffs for conversion into a single summary index. Tariffs are often elicited from the general population by trade-offs in risk or time, in contrast to rating scales such as the visual analogue scale (VAS). Based on the patient’s reported health profile the tariff is applied to obtain utility values, often bounded between 0 (death) and 1 (full health). The revealed valuation for a health state is said to represent the utility weight, which in turn is used to estimate the quality adjusted life year (QALY), i.e. the utility weight times the life years (or survival) in that health state [3].

The outcomes from an investment in stroke care can then be evaluated against other alternative investments that affect health in the society. The most common instruments are EQ-5D [4], Short Form 6D (SF-6D), the latter derived from the generic health profiles SF-12 or SF-36 [5,6], Health Utilities Index Mark 3 (HUI3) [7], Quality of Well-Being scale (QWB) [8], and Assessment of Quality of Life (AQoL) [9]. However, few registries have included any preference-based HRQoL instrument. The Australian Stroke Clinical Registry and Riks-Stroke (RS), the Swedish Stroke register, have used the EQ-5D at least occasionally in their 3-month follow-up questionnaires [2,10]. A potential solution to this lack of generic preference-based measures could be a method called “mapping” or “cross walking” which has gained interest during the last decade [11-13]. In short, this technique is based on estimating the relationship between preference-based and descriptive measures through regression-based transformations, called “transfer to utility” [14]. This has also been done for stroke, but it is limited to validated functional or dependence status and non-preference based generic HRQoL instruments [13-17].

The purpose of this study was to explore the possibility to develop an algorithm which estimates the correlation between the EQ-5D and some outcome measures relevant for stroke and stroke registers, not restricted to validated instruments. If successful, it would give an indication of the usability of the mapping methodology where no validated HRQoL instruments are available. The same methodology could then be applied to other stroke registers as well. It would also enable the translation of historical registry data into EQ-5D weights, allowing for analyses of previous stroke care developments in terms of QALY gains or losses.

## Method

### Data

Patient level data was taken from Riks-Stroke, a national quality register for acute stroke in Sweden. It was established in 1994 to improve and to ascertain a uniform quality of care across geographic areas in Sweden. It covers all of Sweden’s 78 hospitals that admit patients with acute stroke, and validations have shown that at least 85% of all hospital admissions for acute stroke are included in the register [2]. Data collection in RS includes patient characteristics, patient living conditions, process- and outcome variables. Data is collected during the acute phase of the stroke and at a 3-month follow-up by questionnaire, which includes patient-reported outcomes and rehabilitation after stroke. Case record forms are available on the RS website http://www.riks-stroke.org. Patient benefits are measured in terms of survival, activities of daily living (ADL) dependency and living conditions, satisfaction with health care, low mood and general health.

We used two cross-sectional RS data sets from two different periods with patients who had experienced their first haemorrhagic or ischaemic stroke (ICD10: I61, I63 and I64) at age 18 or above. The first data set was originally collected by Lindgren et al. in 2006 for estimating utility loss and indirect costs after stroke at six centres [18]. They analysed utilities at 3, 6, 9, and 12 months after the first stroke among patients aged 18 to 75. However, the data collection did not have an upper age limit, and therefore we were able to retrieve 130 patients who responded to the 3-month follow-up questionnaire, i.e. an additional 73 patients compared with the 57 observations analysed in their study.

In April through December 2009 we performed an additional data collection among 772 consecutively recruited patients at 65 hospitals at three months after the index event (ethical approval: Dnr 95-023, Umeå, Sweden). The data collection procedure was the same as in 2006. The aim was to have complete data from both EQ-5D and RS questionnaires for 400 patients. This would leave us with more than 500 observations in total, which we deemed reasonable on the basis of a review by Mortimer et al. where the median sample size was around 500 [11]. The two data sets were pooled to provide two randomly split samples – one for model estimation and one for model validation. Patients with incomplete, or lacking, EQ-5D index data were excluded.

Both data sets consisted of RS’s regular acute phase and 3-month follow-up questionnaires. Data from the acute phase included age, sex, haemorrhagic or ischemic stroke, risk factors, admission to a stroke unit (i.e. a specialised stroke team with dedicated premises), stroke severity at hospital admission (according to the Reaction Level Scale -85 (RLS) ranging from fully conscious to coma [19]), amongst others. The follow-up questionnaire captured information on the patients’ living arrangement, personal ADL (activities in daily living; mobility, toileting, dressing), cognitive and communicative problems (speech, reading, writing), swallowing problems, self-reported depression, perceived general health, and satisfaction with the care given. For patients unable to respond themselves, care givers were asked to complete the questionnaire on behalf of the patients and information on proxy response was included.

In addition, the EQ-5D visual analogue scale (VAS) has been included in the RS 3-month follow-up questionnaire since 2007, whereas the EQ-5D index was occasionally used in years 2006 and 2009. The EQ-5D has five dimensions to capture the HRQoL (mobility, self-care, usual activities, pain/discomfort, and anxiety/depression), each with three levels of severity (no problems/some or moderate problems/extreme problems), which allows for 243 unique health states [3]. The UK social tariff [20] was used to calculate the EQ-5D index in this study.

### Selection of mapping variables

In order to identify variables in the RS questionnaire that would be appropriate to map with, we excluded all variables related to health care resource in order to avoid geographical and temporal differences in health care resources, e.g. access to stroke unit, rehabilitation and institutional living, etc. The few variables in the RS questionnaire that were conceptually equivalent to the domains of the EQ-5D index questionnaire were:

•Assistance needed with toilet (yes, no)

•Assistance needed with dressing/undressing (yes, no)

•Restricted mobility (none (reference), indoors but not outdoors, both)

•General health (very good (reference), fairly good, fairly bad, very bad)

•Low mood (never (reference), sometimes, often, always)

These outcome measures represent important dimensions for stroke patients [2]. They cover both objective functional outcomes and self-assessed health outcomes after stroke. We also controlled for if the questionnaire had been answered by a proxy, i.e. a next of kin or health professional, in order to capture HRQoL variables among patients with cognitive or communicative problems [21]. Although they may not be as reliable as patient self-assessment, discarding these responses means that we would not have any indication on the HRQoL for patients unable to respond for themselves – maybe the patients with the greatest need. It has been shown that pain, emotion and social functioning are those domains with the lowest agreement between proxy and patient, and the proxy has a tendency to overestimate the impairment. As long as the bias is consistent in how proxies assess these domains, we would make the same error for all cohorts [21]. By including proxy responses we would therefore be able to study the change in improvement over time at the group level for patients who had experienced a severe stroke.

All variables were transformed into dummy variables with the category corresponding to “no disability” as the reference. The reference in the proxy variable was if the patient had answered in writing, with assistance of kin or health care personnel, per telephone or at a follow-up visit. Signs of multicollinearity (volatile coefficients and sign changes) were considered when adding variables and categories to the model. The correlation matrix of coefficients and variance inflation factors (VIF) were analysed [22].

### Model selection and validation

We used three different regression models to estimate the association between EQ-5D weights and independent factors: ordinary least square (OLS), Tobit, and censored least absolute deviation (CLAD) regressions, the two latter to account for the ceiling effect at 1. We estimated the OLS parameters using robust standard errors to adjust for heteroscedacity. This would not affect the estimated parameter values but the variance, and therefore the inference.

However, the OLS has the disadvantage that it does not capture the ceiling effect at 1 in the EQ-5D weights. This ceiling effect results in a censored dependent variable, indicating that values higher than a certain threshold were not measured, which in the case of EQ-5D utilities is 1. Therefore we also included Tobit and censored least absolute deviation (CLAD) models, which allows for censored dependent variables and censored the predicted values at 1. However, the Tobit model has been criticised for generating biased estimates if the assumptions of normality and homoscedasticity are violated [23]. Although the CLAD does not rely on these assumptions, it strives to minimise the absolute deviation of the median, which may not be entirely relevant for applications where the resulting utilities are used for economic evaluations [24].

For prediction accuracy we used the mean absolute error (MAE), i.e. the mean of the absolute prediction error between the observed and estimated individual EQ-5D weights, and mean square error (MSE), the latter taking into account both the bias and variation of the error. In addition, we calculated the percentage of individual observations for which the AE was <0.05 or <0.10 [12]. All statistical analyses were performed in STATA/IC 11.2 (StataCorp, College Station, TX, USA).

## Results

We obtained 544 observations with complete EQ-5D index and clinical variable responses at 3 months after the index event, whereof 105 and 439 were collected in 2006 and 2009, respectively. Statistically significant differences in means and proportions between the samples (P < 0.05) were found in age, the proportion of patients admitted to a stroke unit, ischemic strokes (ICD-10: I63) and proxy responses, Table 1. These differences were considered sample selection bias, apart from admission to a stroke unit, as these units were developed during the period. Responders to the 2009 sample with complete data (57%) were three years younger (74 *vs*. 77), were more frequently fully awake at admission (RLS1: 90% *vs*. 80%) and had fewer recurrent strokes (26% *vs*. 37%) compared to incomplete data or non-responders, all statistically significant at P < 0.05. Apart from patients with atrial fibrillation, there was no statistical difference between the estimation and the validation sets, Table 1.

**Table 1:** Patient characteristics and differences between the 2006/2009 samples and the pooled estimation/validation sets

	**2006 sample (n = 105)**		**2009 sample (n = 439)**		**P**	**Estimation set (n = 272)**		**Validation set (n = 272)**		**P**
	**Mean/Proportion**	**SD**	**Mean/Proportion**	**SD**		**Mean/Proportion**	**SD**	**Mean/Proportion**	**SD**	
Age	71	13	74	11	0.048	73	12	73	11	0.934
Male sex	55%	0.50	54%	0.50	0.785	52%	0.50	56%	0.50	0.391
RLS 2-8	6%	0.23	10%	0.30	0.199	9%	0.29	9%	0.29	1.000
Stroke unit	45%	0.50	75%	0.43	0.000	69%	0.46	69%	0.46	0.831
Ischemic stroke	80%	0.40	89%	0.32	0.019	87%	0.34	87%	0.34	0.899
Previous stroke	17%	0.38	26%	0.44	0.063	24%	0.43	24%	0.43	0.862
Atrial fibrillation	20%	0.40	26%	0.44	0.234	28%	0.45	21%	0.41	0.048
Diabetes	13%	0.34	19%	0.39	0.211	19%	0.40	16%	0.36	0.283
Hypertension	51%	0.50	58%	0.49	0.219	54%	0.50	60%	0.49	0.150
Smoker	20%	0.40	14%	0.35	0.120	16%	0.37	15%	0.35	0.637
Proxy response	2%	0.14	9%	0.29	0.011	10%	0.29	6%	0.24	0.153
EQ-5D index	0.57	0.42	0.61	0.38	0.258	0.61	0.41	0.60	0.36	0.756
EQ-5D VAS	62	24	66	23	0.104	66	24	64	22	0.492

Note: *SD* standard deviation, *RLS* 2-8 sluggish or comatose at hospital admission; *VAS* visual analogue scale.

The numbers of complete responses to the EQ-5D VAS were only 194 and 211 in the estimation and the validation sets, respectively. Patients generally rated their utility higher with EQ-5D VAS, and with a lower coefficient of variation, than with EQ-5D index valued by the UK preference weights. The lower variation stemmed in part from the narrower response interval ranging from 0 to 100 with the VAS, whereas the lowest and highest utility values attainable with 5D using the UK tariff were -0.594 and 1, respectively.

All three models indicated that many of the selected variables were important determinants for stroke patients’ perceived utility, Table 2. The parameter estimate for “toilet assistance” was not significant in any model. The upper confidence intervals for the “proxy response” coefficient in both the OLS and Tobit models were close to zero (the threshold for statistical significance) whereas in the CLAD model it was clearly not statistically significant and with a small parameter value. In addition, the CLAD model indicated that neither the perceived “moody sometimes” nor the “no mobility” variables were meaningful explanatory variables. As expected, the maximum attainable value differed between models, i.e. the maximum utility (OLS: 0.90; Tobit: 1.00; CLAD: 1.00). Although all coefficients were consistent with respect to the sign regardless of the model specification, there were differences in the size of the coefficients. Especially the variables concerning general health were sensitive to the choice of model with relative differences in coefficients around 50% between the OLS and CLAD models. The goodness of fit was not comparable between models due to differences in R^2^ estimations, but the OLS R^2^ of 0.72 indicated that 72% of the variance was captured in the selected variables. Tests for multicollinearity between coefficients indicated a correlation only between “Toilet assistance” and “No mobility” (0.744) and a VIF of 4.94. Still, we considered multicollinearity less of a problem as we were mainly interested in fitting a predictive and not a descriptive model.

**Table 2:** Regression model statistics with EQ-5D weights as the dependent variable

**Regression model**	**OLS**			**Tobit**			**CLAD**		
	**Coef.**	**95% CI**		**Coef.**	**95% CI**		**Coef.**	**95% CI**	
		**Lower**	**Upper**		**Lower**	**Upper**		**Lower**	**Upper**
Constant	0.902	0.843	0.961	1.068	0.970	1.167	1.000	0.997	1.000
Toilet assistance	−0.151	−0.364	0.062	−0.138	−0.325	0.048	−0.179	−0.418	0.061
Dressing assistance	−0.187	−0.301	−0.072	−0.218	−0.362	−0.074	−0.247	−0.446	−0.048
Indoor mobility only	−0.191	−0.266	−0.116	−0.230	−0.344	−0.117	−0.152	−0.240	−0.064
No mobility	−0.363	−0.570	−0.156	−0.393	−0.580	−0.206	−0.281	−0.587	0.025
General health fairly good	−0.055	−0.126	0.016	−0.129	−0.233	−0.024	−0.121	−0.229	−0.014
General health fairly bad	−0.155	−0.263	−0.046	−0.233	−0.367	−0.099	−0.237	−0.367	−0.107
General health bad	−0.246	−0.463	−0.028	−0.303	−0.529	−0.077	−0.370	−0.651	−0.088
Moody sometimes	−0.074	−0.131	−0.017	−0.137	−0.215	−0.060	−0.107	−0.255	0.011
Moody often	−0.208	−0.354	−0.062	−0.274	−0.413	−0.134	−0.223	−0.400	−0.045
Moody always	−0.327	−0.503	−0.150	−0.387	−0.610	−0.163	−0.471	−0.788	−0.154
Proxy response	−0.132	−0.271	0.007	−0.141	−0.280	−0.001	−0.032	−0.266	0.202
R^2^ / Pseudo R^2^	0.724			0.657			0.486		

Note: OLS = ordinary least square; CLAD = censored least absolute deviation; Coef = coefficient; CI = confidence interval.

All three models over-estimated the mean utility, Table 3. The OLS model generated a mean closest to the observed mean and had the smallest MAE and MSE. However, this lower variation in the OLS model came with an inability to predict the minimum and maximum values as good as the Tobit and CLAD models. The range of predicted health states was greatest with the Tobit model, but it also generated the highest predicted errors. The CLAD model provided the best predictions when limiting the error tolerance to 0.05 followed by the OLS model. When extending the tolerance to <0.10, the OLS and Tobit models performed best. Note that an absolute error of 0.10 was more than half the mean MAE (average 0.17 for all three models) but still less than 50% of the observations (average 43% for all three models), indicating a long tail of absolute errors.

**Table 3:** Validation of models on the validation sample, observed and predicted EQ-5D weights

**Model**	**Mean**	**SD**	**Min**	**Max**	**MAE**	**MSE**	**AE < 0.05**	**AE < 0.10**
Observed (n = 272)	0.599	0.364	−0.594	1.000	n/a	n/a	n/a	n/a
OLS	0.639	0.287	−0.293	0.902	0.167	0.056	21%	44%
Tobit	0.682	0.324	−0.328	1.000	0.173	0.061	20%	45%
CLAD	0.659	0.306	−0.300	1.000	0.169	0.059	24%	41%

Note: *SD* standard deviation; *MAE* mean absolute error; *MSE* mean square error; *AE* absolute error; *n/a* not applicable; *OLS* ordinary least square; *CLAD* censored least absolute deviation.

**Figure 1:**
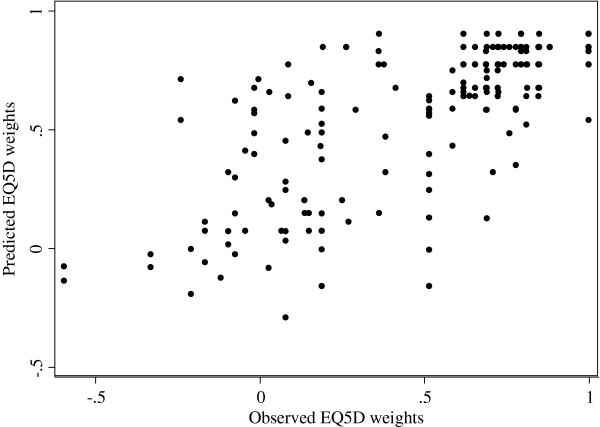
Plot of observed vs. predicted EQ-5D weights (OLS).

**Figure 2:**
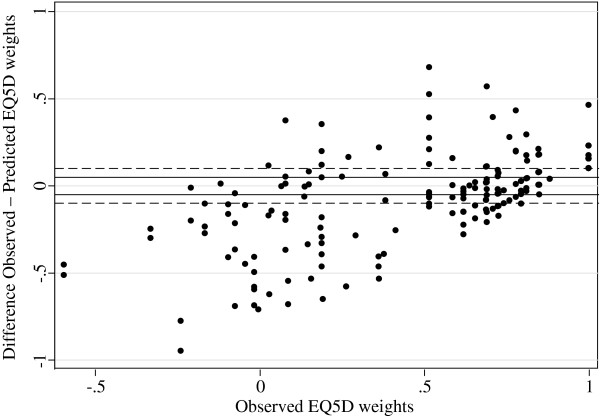
Plot of EQ-5D prediction errors from OLS-model vs. observed EQ-5D weights.

Figure 1 presents a scatterplot of the observed and predicted EQ-5D weights of the validation sample estimated with OLS (the other models produced similar plots). Ideally these observations should be located along a diagonal line from -0.594 to 1. However, as seen in Figure 2, the prediction errors formed clusters above and below the value of 0.5 (P < 0.05 for all models). Below the value of 0.5, the predictions had higher variation and overestimated the utility by 0.26 units on average, whereas the predictions above 0.5 had lower variation and neither over- nor underestimated the EQ-5D weights. In fact, mean error in EQ-5D ranges <0, 0.00 to 0.24, 0.25 to 0.49, 0.50 to 0.74, and 0.75 to 1 were -0.31, -0.19, -0.15, -0.01 and 0.10, respectively. This would indicate that the models provide better predictions for better than for worse health states.

## Discussion

We developed an algorithm for translating variables used for health care quality assessment into utility weights suitable for comparing developments in Swedish stroke care with other medical conditions. Data for 544 patients 3 months after their first stroke were split into an estimation and a validation set for assessing three different statistical models: OLS, Tobit and CLAD. Several of the mapped variables were close to the domain questions in the EQ-5D which could explain the good fit in all the specified models. The results also indicated that the variable selection and parameter estimates differed with the model specification. Each model had its own advantages/disadvantages in terms of predicting the EQ-5D index weights, and all overestimated the mean weight. The OLS provided a mean closest to the observed (mean error 0.04 QALY) but had the most compressed variation resulting in an inability to predict minimum and maximum values correctly.

Our results are in line with what Brazier et al. reported in a recent review of mapping studies [12]. They found that the mean error in 119 prediction models ranged from 0.0007 to 0.042 and MAE between 0.0011 and 0.19 (0.167 in our OLS model). It was also reported that most studies had lower variance in the predicted values than in the observed and that there was a tendency for prediction errors to be greater at the lower end of the scale (worse health).

The EQ-5D instrument has been shown to capture the HRQoL aspects in stroke patients well, especially for disability and ADL, although not as sensitive as disease specific instruments [25,26]. However, long-term effects mainly affect mental dimensions of HRQoL due to adaptions and coping strategies to physical disability [15,27-29]. This could indicate that the same algorithms may not be applicable to samples at other points in time than 3 months, or for samples with other symptoms than in our study.

Compared with the average stroke patient in 2009 reported by Riks-Stroke, fewer patients in our sample were admitted to a stroke unit (75% vs. 87%), ischemic strokes may be slightly overrepresented, more patients were fully awake at admission (RLS 1; 90% vs. 82%) but were approximately the same age [30]. However, for the purpose of this study, full representativeness may not be that important as we analysed the relationship at an individual level for predictions at a group level.

We chose to include responses provided by proxies for patients unable to answer the questionnaires by themselves although proxy response reliability is lower [21]. Indeed, we found that proxies rated the utility lower even when controlling for mood. We argued, however, that as long as we would make a *consistent* error in all cohorts, this potential bias would be outweighed by the gain from including patients with greatest needs, e.g. who have experienced a severe stroke.

We based our regressions on utility weights using the UK social tariff developed by Dolan in the absence of a corresponding Swedish tariff of health state utility weights [20]. A multinominal logistic regression (MLogit) would estimate the actual index responses, i.e. the health states, from the explanatory variables and thereby allow for applying a country specific tariff. In addition, this technique would eliminate the problem with the ceiling effect at 1 [11]. However, as the data we used is unique to Sweden we did not consider this specification. In addition, Rivero-Arias et al. showed that the difference in predicted utility did not differ very much with OLS predictions when mapping mRS to EQ-5D, although the Mlogit provided a better fit for worse health states [16]. However, as Mortimer and Segal point out in a comprehensive review, individual prediction errors of EQ-5D weights may not present a problem for group level analysis [11].

Still, it is necessary to reflect on the implications of the slight under-estimation of the predicted mean, the lower variability, and the unevenly distributed prediction errors across the scale. The unevenly distributed prediction errors limit the use of mapped utility to observations closer to the interval where the regression errors are smaller, i.e. closer to the mean in our study. In other words, the overestimation of more severe health states indicates that the methodology may not be suitable for subgroup analyses based on severity. Biases may differ not only depending on the choice of model and which preference-based measure is mapped, but also between the conditions modelled [31]. As utilities often are used in economic evaluations, which analyses incremental costs in relation to gained utility, the properties of the denominator can have a great impact on the resulting incremental cost-utility ratio. In fact, the use of different mapping models can result in different reimbursement decisions [31,32]. Consequently, utilities estimated through mapping may have lower ranking among health technology assessment agencies [33]. Utility weights derived from mapping studies should therefore be used with caution and seen as a second alternative to primary data when used for cost-utility analyses [12]. Still, the ability to estimate utilities from data captured for other purposes can provide important information in the lack of primary data, e.g. assessment of health care development over time or for sensitivity analysis in economic evaluations. Although other registries record different variables than included in our study, the mapping methodology could be a source of valuable information.

## Conclusion

This study indicates that mapping health-related outcome variables into preference-based utilities can be done with fairly good precision even though the mapped variables are not part of any validated instrument. Although the precision depends on the ability of the mapped variables to capture the HRQoL domains relevant among stroke patients, the choice of statistical model, the severity of the patients, and the analytical purpose of the predicted utility, we believe that the mapping methodology could be used by other health care quality registers to evaluate the care development in terms of QALY gains or losses. In our sample the OLS model produced the best fit for predicting the utility mean.

## Consent

Written informed consent was obtained from the patients for publication of this report.

## Competing interests

The authors declare that they have no competing interests.

## Authors’ contributions

OG participated in the design, data analysis, interpretation of results and drafted the manuscript. ME participated in the design, provided statistical input and edits and revisions to the manuscript. E-LG supervised and participated in the design, interpretation of the results, and provided edits and revisions to the manuscript. All authors read and approved the final manuscript.
